# Supplementary material for: Blockade of CDK7 Reverses Endocrine Therapy Resistance in Breast Cancer
Source: Int J Mol Sci. 2020 Apr 23;21(8):2974. doi: 10.3390/ijms21082974 (PMC7215326; doi:10.3390/ijms21082974)
Supplement: Supplementary file 1 [file ijms-21-02974-s001.pdf]

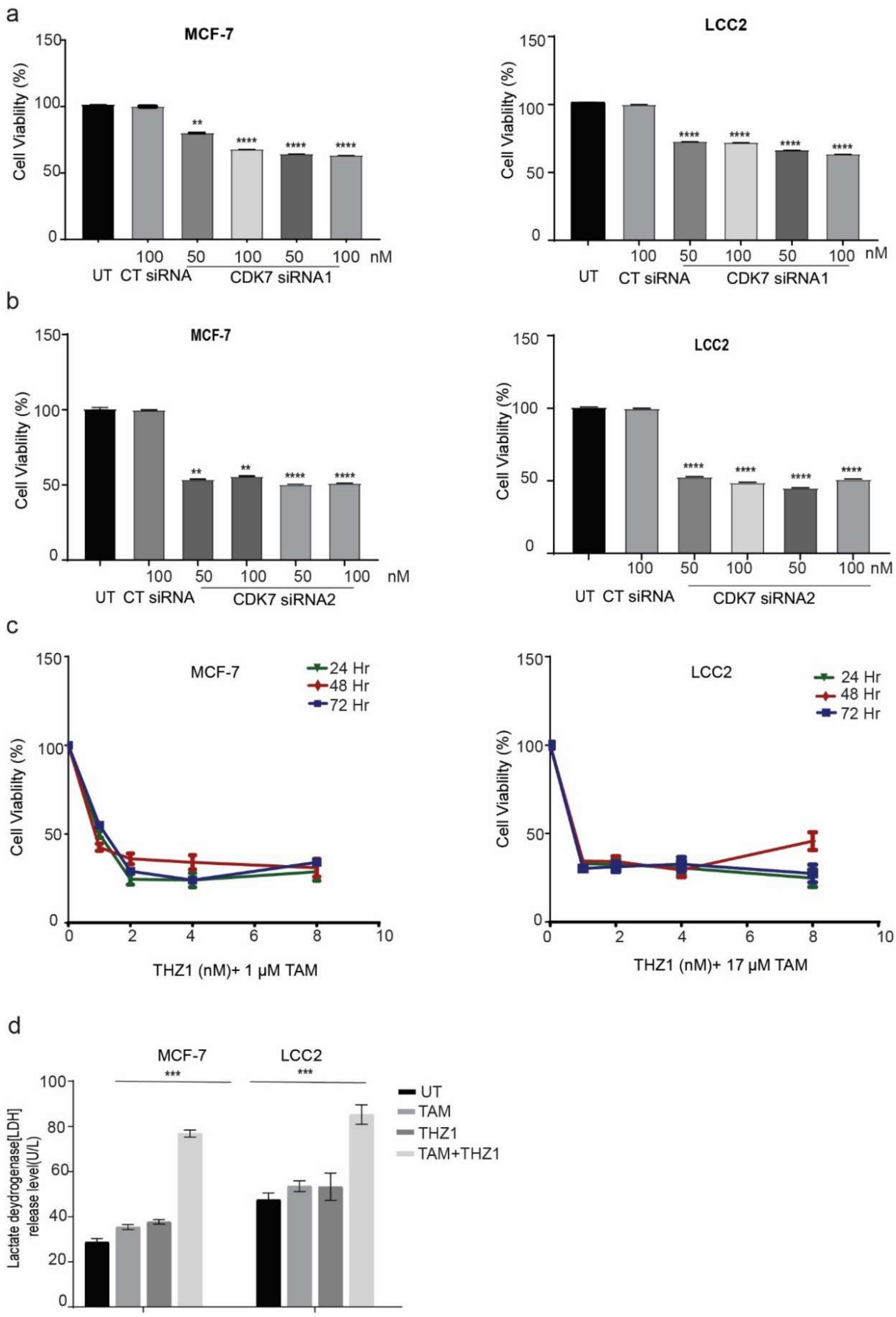

Supplementary Figure S1. Cytotoxicity after treatment of breast cancer cells with CDK7-targeting treatments. **(A)** Viability of MCF-7 and LCC2 cells treated with the indicated concentrations of siRNA-CDK7-1 (25-100 nM). **(B)** Viability of MCF-7 and LCC2 cells treated with the indicated concentrations of siRNA-CDK7-2 (25-100 nM). **(C)** Viability of MCF-7 and LCC2 cells treated with THZ1 (1-8 nM) and 1 or 17 μM tamoxifen (TAM), respectively, after the indicated incubation times. **(D)** Lactate dehydrogenase release levels in MCF-7 and LCC2 cells after treatment with 1 and 17 μM tamoxifen, respectively, and 1 nM THZ1. UT, untreated, CT, control. $^{**}P < 0.01$; $^{***}P < 0.001$; $^{****}P < 0.0001$.

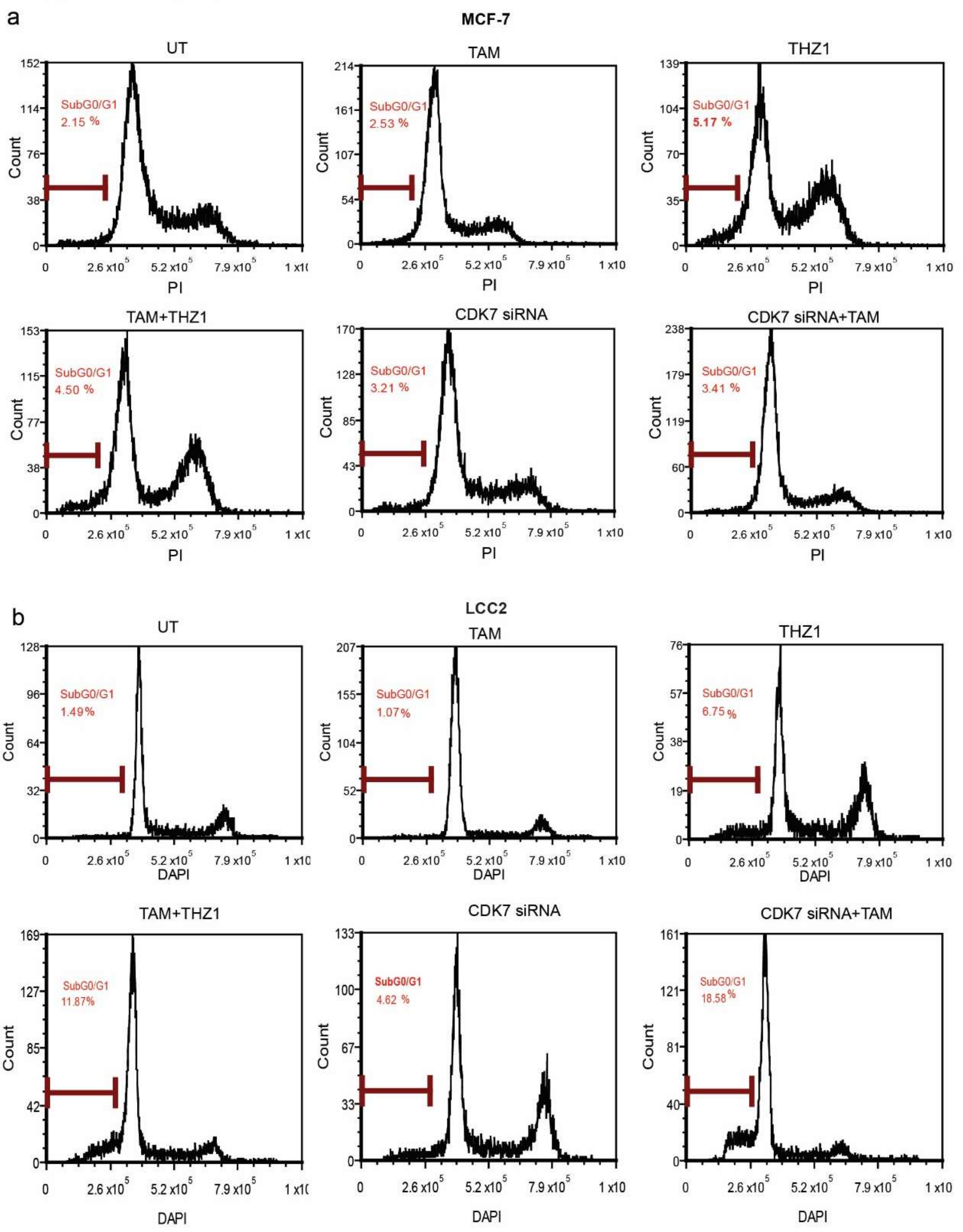

Supplementary Figure S2. Cell cycle analysis of MCF-7 and LCC2 cells. **(A)** Flow cytometry analysis using propidium iodide, MCF-7 cells untreated (UT), CT siRNA 1 μM tamoxifen (TAM) or/and 1 nM THZ1 or transfected with 50 nM CDK7 siRNA or/and tamoxifen. **(B)** Cell cycle analysis using DAPI of LCC2 cells untreated, CT siRNA, 17 μM tamoxifen or/and 1 nM THZ1 or transfected with 50 nM CDK7 siRNA or/and tamoxifen.

a

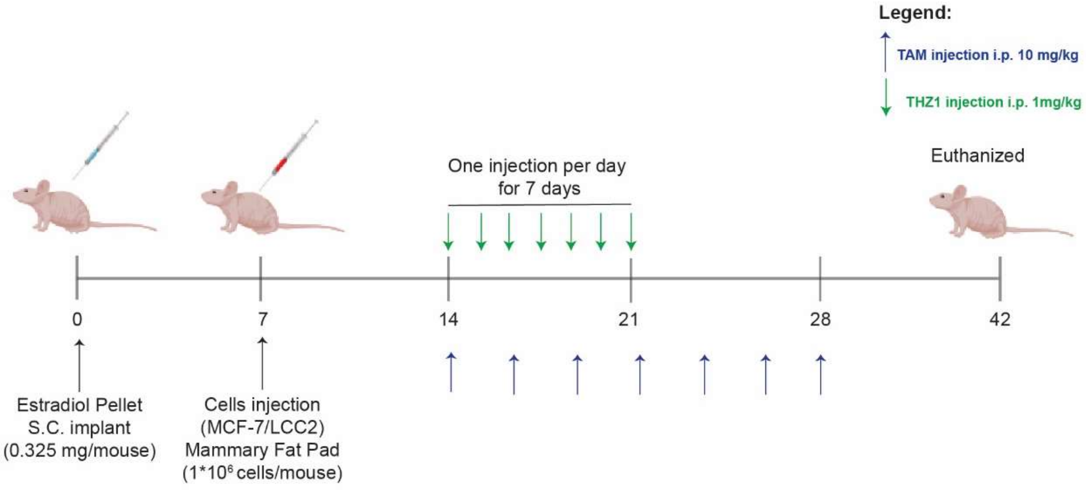

b

Scheme of Treatment		
Group	Number of mice	Treatment
1	10	Untreated
2	10	Tamoxfen (1 mg/100μl)
3	10	THZ1 (10 mg/Kg)
4	10	Tamoxifen (1mg/100μl) + THZ1 (10mg/Kg)

c d

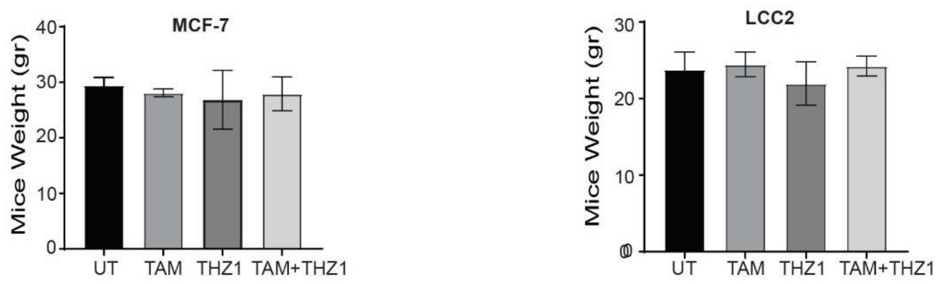

Supplementary Figure S3. In vivo experiments. **(A)** Design of the mouse models of MCF-7 and LCC2 and the treatment plan. **(B)** The treatment scheme for the four groups. **(C and D)** Mouse weight (10 per group) in control untreated (UT), tamoxifen (TAM)-treated, THZ1-treated, and combination-treated groups in mouse models of MCF-7 **(C)** and LCC2 **(D)**.
